# A system for computerised retinal haemorrhage analysis

**DOI:** 10.1186/1756-0500-2-196

**Published:** 2009-09-28

**Authors:** Tariq Aslam, Paul Chua, Matthew Richardson, Praveen Patel, Mohammed Musadiq

**Affiliations:** 1 Moorfields Eye Hospital, London, UK; 2 Princess Alexandra Eye Pavilion, Edinburgh, UK; 3 Dept. of Ophthalmology, University Hospital of North Staffordshire, Stoke-on-Trent, UK

## Abstract

**Background:**

Our aim was to develop an objective computerised system for measuring different types of retinal haemorrhages in differing digital images, for use as a research tool. Despite developing various fully automated systems of retinal haemorrhage measurement we ultimately found user interaction to be necessary to achieve satisfactory validity of segmentation, and developed an interactive system of haemorrhage assessment based on this.

**Findings:**

The Haemorrhage Assessment System (HAS) presented here is an open access interactive program with graphical user interface allowing the ophthalmically trained user to easily delineate different haemorrhage types, optic disc and fovea. The system then automatically calculates a variety of measures, including mean haemorrhage area, total haemorrhage area, centre of mass, distance of centre of mass from disc and fovea, eccentricity and orientation. This paper presents evidence for the validity of HAS by comparison against established software and known results for geometric images.

**Conclusion:**

The system should be of use to experimenters studying the distribution and topography of vitreoretinal haemorrhages who require a means of accurately quantifying an ophthalmologist's gold standard assessment of a digital image.

## Background

Haemorrhages occur when there is extravasation of blood into the retina itself (deep and superficial retina haemorrhage), between the retina and the hyaloid face of the vitreous body (pre-retinal), or into the vitreous gel (vitreous haemorrhage). The colour and the shape of the haemorrhages are determined by the layer or layers of retina that are affected. Haemorrhages in each of these areas have different features of texture, shape, hue, intensity, saturation and edge.

Objective analysis of retinal haemorrhage might be useful in several ways. Firstly, it may be helpful in clinically differentiating conditions, e.g., birth trauma and shaken baby syndrome[1] - current studies are hampered by lack of objective outcome measures. Secondly, quantitative characterization of retinal haemorrhage together with accurate assessment of other biomarkers of the patient may provide insight into the aetiology and pathogenesis of the haemorrhagic condition[2]. Finally, detailed image analysis may help in the grading of retinopathy e.g., retinal haemorrhage in cerebral malaria[3].

Published methods for haemorrhage detection are often focussed on automated detection of intraretinal haemorrhages in diabetic retinopathy images. Gardner et al. [4] employed a back propagation neural network to detect diabetic haemorrhage. The success rate depended on the pre-processing and the number of images used in training. They reported detection rate for haemorrhages as 73.8% for both sensitivity and specificity, compared with the reference standard of a clinical ophthalmologist.

Sinthanoyothin et al[5] utilised a "moat operator" in their image analysis which creates a trough around the lesion and aids segmentation of the image to enhance the hemorrhagic lesions. Recursive region growing segmentation was then used to extract blood vessels and haemorrhage lesions. Sensitivity and specificity for haemorrhage detection were 77.5% and 88.7% respectively. Again this was compared with assessment by a clinical ophthalmologist.

Usher et al[6] employed recursive region growing and adaptive intensity thresholding in conjunction with a "moat operator" similar to Sinthanoyothin et al[5]to extract haemorrhagic lesions. Then, a neural network was applied to classify the images. On a per image basis, sensitivity for detection of any exudates and/or haemorrhage/microaneurysms was 89% and a specificity of 62%.

M. Niemeijer et al[7] proposed a haemorrhage detection scheme based on pixel classification. By using pixel classification, the contrast between the foreground pixels (red lesions and vasculature) and pixels in the background can be improved such that a global threshold can be used to extract all relevant objects. Subsequently, the vasculature was separated from the red lesions. This method was then refined by combining the mathematical morphology as used by Spencer-Frame[8,9] to form a "Hybrid Scheme". The hybrid system reached 100% sensitivity and 87% specificity on a per image basis for haemorrhage, although the sensitivity on a per lesion basis was 30%. Grisan et al[10], Larsen et al[11] and Lee et al[12] have also employed image processing and analysis techniques to test for haemorrhages.

Apart from in diabetes, haemorrhage assessment would also be of use for assessing paediatric non-accidental injuries[13] and other conditions with areas of haemorrhage such as retinal vein occlusions that would benefit from being accurately quantified for clinical or research purposes [2,14]. There are particular problems associated in automated assessment of images of these conditions. Haemorrhages may be present at different levels in the eye and have subtly different physical characteristics within each level. The blood may be in vitreous gel, between vitreous and retina (subhyaloid haemorrhage), at superficial or deep retinal layers. Haemorrhages in each of these areas have different features of texture, shape, hue, intensity, saturation and edge. Vitreous haemorrhage in particular can be extremely variable from barely noticeable to completely obscuring all retinal view. It poses a challenge not only for its own specific quantification but also for that of other forms of haemorrhage given that it obscures retinal clarity. Image quality may be further degraded due to difficulty in obtaining images both in infants and in adults (due for example to cataract or high intraocular pressures causing poor visibility).

All the automated systems for retinal haemorrhage analysis have limited sensitivity and specificity as demonstrated above. A key cause for this is that retinal haemorrhages have high variability in lesion appearance, both intra-image and inter-image, coupled with the fact that the colour content of hemorrhagic lesions is often within the distribution of retinal background pigmentation. Variations in patient ethnicity also cause difficulty in standardization of image processing and analysis settings as retinal pigmentation levels vary. Furthermore, algorithms [5-7,11,12] that use a two-stage segmentation/classification approach needs a very sensitive first stage and very specific second stage.

The gold standard for assessing retinal haemorrhage alluded to in all the above papers is still universally acknowledged to be clinical assessment by retinal specialists. However, there is no open access system designed to allow such specialists to perform this in a way that benefits fully from computer technology. We present software that allows this gold standard clinical assessment of images to be done in a specially designed computing environment and adds high level computing calculations that would otherwise have been prohibitively complex.

## Algorithms

The Haemorrhage Assessment System (HAS) was designed and written using MATLAB^© ^version 14. It has been compiled for use with Windows XP providing MATLAB^© ^installer is used. There is a greater access to viewing options, however, if the uncompiled version of the program is run within the MATLAB^© ^runtime environment. The program was written by the first author (TA).

We initially developed fully automated systems of analysing haemorrhages. The most promising involved an initial pre-processing sequence of background removal and adaptive histogram equalisation followed by conversion to hue, saturation and intensity (HSI) colour map modes. Specific thresholds for hue, saturation and intensity components were used to highlight central seed components of the haemorrhage. These were grown into mask elements that had less demanding thresholds. We devised a program that allowed us to investigate and adapt these thresholds using separate sliders for hue, saturation and intensity for both seed and mask. For individual images these could be altered to produce segmentation results that were acceptable for retinal haemorrhage detection. However there were no settings that provided excellent sensitivity and specificity for segmentation with broad validity across varied images with different forms of haemorrhage. Published reports are ultimately limited in their sensitivity and specificity of haemorrhage detection as described above and do not include eyes with highly varied forms of haemorrhage including vitreous haemorrhage[11,15]. We ultimately found that user interaction was required to achieve higher levels of validity for segmentation of different types of haemorrhage in varied patient groups.

The HAS program presents a simple graphical user interface from which the user first loads the image to be analysed. The software allows the option of viewing a processed version of the image in which haemorrhage is more easily visible to the user. The user is required to delineate the optic disc and fovea. The software then requires the user to specify what region he requires to be assessed. This may be done by delineating it manually. Alternatively, the user can choose to assess an automatically segmented area based on the commonly used constant of numbers of disc diameters away from optic disc or disc diameters away from the fovea. The user must then carefully manually mark the boundaries of each type of haemorrhage. When all haemorrhages are marked to the satisfaction of the user, the HAS software performs calculations on these valid segmented areas. The total area of each type of haemorrhage is calculated. The centre of mass of the average of each area type is calculated along with its distance from the centre of the disc and from the fovea. The total values for all haemorrhages combined are also assessed in this way. The 'eccentricity' of the haemorrhagic mass is also calculated-this term alludes to the deviation from circularity, with 0 being a perfect circle and 1 being a straight line. Finally, 'orientation' is calculated and is the angle in degrees between the x axis and the major axis of the ellipse that has the same second moments as the haemorrhage region. The area values are provided both in pixels and disc area units.

Many commercial software packages can perform basic measurement tasks from images. Such software can calculate the area of regions of interest circled by the user. However, the software would be tedious and prohibitively slow to use to calculate multiple haemorrhages at different levels and offers no automatic pre-processing step of background removal with adaptive histogram equalisation. Furthermore, advanced calculations performed by HAS are not available, such as centre of mass, automated calculation of distances from disc and fovea, eccentricity and orientation, nor the facility to assess only set areas of an image based on the constant of optic disc diameter.

## Testing

There should be no doubt that an area marked out on a digital image by an ophthalmology specialist is indeed a retinal haemorrhage - His assessment is the current gold standard and no automated computer algorithms can compete. The HAS acts to provide technical support to this process by allowing the measurement of haemorrhagic areas marked out to be easier, quicker and ultimately much more informative. The testing of HAS focuses therefore on proving that once outlined, the mathematical values of size attributed to these areas are indeed correctly assessed. For this, the appropriate test is to first allow a specialist to measure several areas with HAS. The same areas are then delineated with an established (albeit non-specialised) scientific program. Although such measurements would take longer to complete and give less detailed statistical information than HAS, they should provide values of area that correspond.

In fact, the HAS program was tested by running analyses on a random selection of images of retinal haemorrhages using both the HAS system and a well established scientific image analysis software tool, Image J (1.23p, ). The Image J software used could not fully replace the HAS system as it is deficient in key areas that are done automatically by the HAS system. Firstly it does not pre-process the retinal image for ease of assessment. It does not make important automated calculations such as total haemorrhagic areas or eccentricity. Finally it does not perform functions for calculating centre of optic disc and thus does not provide statistics describing distances from haemorrhages to optic disc and to the fovea. However, Image J software is able to simply measure the area of any image region that is first delineated by the user. Using this function multiple haemorrhages could be separately calculated then added together by the user to recreate the basic area measures of the HAS. The areas could be measured and total haemorrhage area could be calculated by the user and compared to the HAS output for the same images.

Ten assorted haemorrhagic images and combinations of haemorrhage were measured using both systems and compared to ensure calculations by the HAS system were correct. There would not be perfect equality between the two assessments as variations would be expected from imperfect user delineation of boundaries of haemorrhage. We would expect there to be a broad agreement in the two measures if the HAS software is correctly measuring the areas that are being marked by the specialist. Differences between the HAS and Image J areas should not show evidence of bias and there should be no significant differences between findings from the two measures. This was tested using a paired t-test statistic. Sample size calculation was done for a β = 80% and α = 0.05. Expected error from human variation in delineation of same boundary with same program twice from pilot study was around 2% and standard deviation of difference of pixel size between paired samples was 413 in the pilot data assessed. This equated to a small sample size requirement of 5.6 images and 10 were actually assessed in the study. Each image had the same areas of haemorrhage measured by both the Image J and HAS system. Although we would expect some differences due to human variation, there should be no significant difference. These areas are presented in Table 1. A paired T-test carried out on these two groups was performed using SPSS. This gave a value of significance of p = 0.907. Thus no significant difference was found between the two groups.

**Table 1:** 

**ImageNumber**	**Image J Size** **(pixels)**	**HAS Size** **(pixels)**	**Percentage Error (%)**
1	67148	69035	2.81
2	34452	35287	2.42
3	5114	5271	3.07
4	61756	62754	1.62
5	179695	174955	2.64
6	156022	158597	1.65
7	99035	97492	1.56
8	7271	7339	0.94
9	7073	6812	3.69
10	124239	125040	0.64
			
**Mean**	74180.5	74258.2	2.10
			
**Significance**			**p = 0.907**

Other statistical analyses performed in the HAS program such as centre of mass, eccentricity, distance from fovea and disc and orientation of highlighted shapes utilise the widely used and well tested algorithms of the image processing toolbox of MATLAB^© ^software. These calculations are unlikely to be at fault if correct templates of area of interest are input and have been extensively tested in the Matlab runtime environment. However, validity of these calculations was, in addition, checked by measuring standard geometric shapes for which the outcome of these complex calculations could easily be predicted. For example, a perfect circle should have an eccentricity of zero and line segment should have an eccentricity of one. Centre of mass of regular symmetrical shapes could similarly be easily predicted. Results of these analyses are shown in Table 2.

**Table 2:** 

	**Expected value**	**HAS Value, including human tracing error**
**centre of mass of square**	135 × 289	134.5 × 288.5
	300 × 300	300 × 300
**centre of mass of circle**	138 × 133	137 × 132
	145 × 315	146 × 316
**eccentricity of line shape**	1	0.999
		
**eccentricity of near circular shape**	<0.2	0.106

There is an excellent level of accordance between Image J calculated areas and the HAS system and excellent accordance between expected values for complex statistical analysis of particular geometric shapes and those values returned by HAS.

## Implementation

Project name; Haemorrhage Assessment System

Project homepage; 

-All instructions and required files available from this site.

Availability and requirements; Open availability. Requires Windows XP/MATLAB^© ^runtime environment preferred. *The AMD ACML BLAS is not supported on MATLAB 7.3 (R2006b) and on previous releases of MATLAB on the Windows platform thus this program will not run on machines with AMD processors unless the Matlab runtime environment is available*.

Operating System. Windows XP/Vista

Programming language; MATLAB^©^

Restrictions for non academics; for academic use only

The user is guided through the program by a graphical user interface with a column of function buttons on the left side of the screen that should be depressed in turn from top to bottom. Pressing the top button allows the relevant image to be loaded onto the screen. The next 'Process image' button initiates a sequence of processing steps that results in an output image in which areas of haemorrhage are highlighted more clearly than the original image - This step may take a few minutes to complete, but allows the user to toggle between this processed image and the original before deciding which to continue with. After pressing the following button, labelled 'Define region of interest', the user must click the left mouse key around the total area of image that is relevant to the study. A final right mouse key click signifies user segmentation of the relevant area is complete. As a final pre-processing step the user clicks on the 'define disc/fovea' button and is asked to manually delineate these areas. This allows for the option of analysing only within a certain number of disc diameters around the fovea or disc. This is subsequently done, if required, by setting the slider to the appropriate number of disc diameters and then pressing the button marked as measuring 'diameters around disc' or 'diameters around fovea'.

After this the program is ready to accept user input of areas of haemorrhage. To indicate areas deemed as haemorrhage, the user presses the button corresponding to the haemorrhage type before manually delineating the region in the same way as the initial region of interest was outlined. This is repeated for all haemorrhages before the button marked 'Calculate' is pressed. A separate window shows the results of the calculations performed, separated into haemorrhage type and also calculating total haemorrhage indices.

## Conclusion

The HAS system we present helps ophthalmologists quantify their gold standard clinical assessment of haemorrhage. It relies on users to input the haemorrhage borders and thus maintains complete validity for the segments that are being measured. The user is guided through the measurement process via an easy to use graphical user interface. The HAS system ultimately is able to perform complex calculations on the segmented areas that would otherwise be extremely difficult. It is anticipated that the system may be of use for all experimenters interested in studying the distribution and topography of vitreoretinal haemorrhages in retinal and systemic disease.

## Competing interests

The authors declare that they have no competing interests.

## Authors' contributions

TA conceived and programmed the system, devised the validity testing and wrote the paper. PC contributed to testing, interpreting data and critical revision of the paper. MR contributed to testing, data analysis and critical revision. PP and MM advised on presentation and design of the program as well as testing. All authors read and approved the final manuscript.
